# Production of Drug Delivery Systems Using Fused Filament Fabrication: A Systematic Review

**DOI:** 10.3390/pharmaceutics12060517

**Published:** 2020-06-05

**Authors:** Bahaa Shaqour, Aseel Samaro, Bart Verleije, Koen Beyers, Chris Vervaet, Paul Cos

**Affiliations:** 1 Voxdale bv, Bijkhoevelaan 32C, 2110 Wijnegem, Belgium; bart@voxdale.be (B.V.); koen@voxdale.be (K.B.); 2 Laboratory for Microbiology, Parasitology and Hygiene (LMPH), Faculty of Pharmaceutical, Biomedical and Veterinary Sciences, University of Antwerp, Universiteitsplein 1 S.7, 2610 Antwerp, Belgium; paul.cos@uantwerpen.be; 3 Laboratory of Pharmaceutical Technology, Department of Pharmaceutics, Ghent University, Ottergemsesteenweg 460, 9000 Ghent, Belgium; aseelhz.samaro@ugent.be (A.S.); chris.vervaet@ugent.be (C.V.)

**Keywords:** hot melt extrusion, additive manufacturing, fused filament fabrication, fused deposition modeling, 3D printing, drug loading, drug delivery

## Abstract

Fused filament fabrication (FFF) 3D printing technology is widely used in many fields. For almost a decade, medical researchers have been exploring the potential use of this technology for improving the healthcare sector. Advances in personalized medicine have been more achievable due to the applicability of producing drug delivery devices, which are explicitly designed based on patients’ needs. For the production of these devices, a filament—which is the feedstock for the FFF 3D printer—consists of a carrier polymer (or polymers) and a loaded active pharmaceutical ingredient (API). This systematic review of the literature investigates the most widely used approaches for producing drug-loaded filaments. It also focusses on several factors, such as the polymeric carrier and the drug, loading capacity and homogeneity, processing conditions, and the intended applications. This review concludes that the filament preparation method has a significant effect on both the drug homogeneity within the polymeric carrier and drug loading efficiency.

## 1. Introduction

In the 1980s, the process of layer manufacturing was established in Japan, France, and the US [1]. This manufacturing method is similar to the contour maps; i.e., each three-dimensional model is sliced into two-dimensional sections along its height [2]. Subsequently, these layers are 3D printed, one after another, until the complete geometry is produced. One of the first additive manufacturing (AM) machines used stereolithography technology, which was introduced by 3D systems [3]. Since then, several techniques have been introduced such as, selective laser sintering, laminated object manufacturing, fused deposition modeling (FDM™), and others [4]. In the medical field, AM shows great potential [5]. One of the greatest advantages of AM is the capability of producing patient-specific products in a short lead time. An example of 3D printing technologies that have already introduced novel drug delivery systems to the market is “Spritam^®^”. This system was developed by Aprecia Pharmaceuticals and was approved by the Food and Drug Administration in 2015. This drug delivery system is produced using the Zipdose^®^ technology, which relies on a powder bed liquid 3D printing approach [6]. The presented system is a high dosed tablet with rapid disintegration capabilities [7].

FDM™, also known as fused filament fabrication (FFF), is a widely used technology. It has exhibited a considerable reduction in price during the last decade. This technology also has many advantages compared to other 3D printing technologies, such as being user-friendly with minimal post-processing steps for the end-product. In a standard FFF 3D printer, a filament is melt-extruded through a nozzle with a specific diameter that moves in three-dimensional space in a controlled manner to build a 3D geometry. Upon heating, the physical state of thermoplastic polymers changes from solid to semi-solid up to liquid when fully melted. The suitable printing temperature depends on the thermal properties of the polymer and the rheological properties of the melt [8]. 

The filament is usually prepared via the hot melt extrusion (HME) process, which is a well-established technology used for processing polymers. This technology is used for producing different types of products. In principle, HME relies on the change in the physical properties of the materials upon heating. This technology uses a screw-based extrusion system located in a barrel and is driven by a motor. After the explosion of the FFF market in the past decade, the HME technology was heavily used to produce the feedstock material for these 3D printers, which is in the form of a filament with a diameter ranging between 1.75 mm and 3.00 mm. Many process parameters and material characteristics have been explored and investigated to provide the market with different types of materials that can serve various applications [9]. 

Over the past years, numerous reviews have been published that discuss the used of AM technologies for the production of drug delivery systems, such as Hsiao et al. [10] and Korte et al. [11]. Other studies have focused on FFF 3D printing technology, such as Araujo et al. [12] and Mathew et al. [13]. A recent review by Azad et al. [14] discussed various polymers used in such processes and the material properties that should be considered when developing a drug-loaded device. However, none of these reviews or studies provided a detailed discussion of the various techniques and methods that are used for producing a drug-loaded filament. As these techniques vary among researchers, the quality of the produced filament greatly depends on the method used. Additionally, the loading efficiency and homogeneity of the drug within the filament will also greatly affect the efficacy of the produced drug delivery system. Thus, this systematic review focuses on discussing the various drug incorporation approaches into a carrier polymer for producing filaments used for the FFF 3D printing process. Moreover, polymer and drugs that were used in these studies are summarized, along with their intended application. 

## 2. Methods 

This systematic review was based on the PRISMA guidelines [15]. Two electronic databases (WebofKnowledge and PubMed) were used to survey the literature. The keywords selected for this review were divided into two parts. First, fused filament fabrication, fused deposition modeling, FDM™, and FFF; the most used terms that refer to the AM technology under investigation in this review and the most widely used acronyms for this technique. Secondly, drug delivery and drug loading; these two keywords were selected to limit the results from publications related to drug loading applications. This was required as the FFF process has a variety of applications in many different fields such as aerospace, architecture, and product development. 

The search phrase in the WebofKnowledge was “(TOPIC: (fused filament fabrication) OR TOPIC: (fused deposition modeling) OR TOPIC: (FDM) OR TOPIC: (FFF)) AND (TOPIC: (drug delivery) OR TOPIC: (drug loading))” and in the PubMed was “(((drug delivery) OR drug loading)) AND ((((fused filament fabrication) OR fused deposition modeling) OR FDM) OR FFF).”

In this review, a selection of criteria was designated for determining which articles were to be included. These were (1) the drug should be incorporated into the polymer’s matrix prior to 3D printing, (2) carrier polymers should be a thermoplastic polymer, and (3) 3D printing technology used should be FFF technology. Additionally, only original research articles were included, and other publications such as conference papers, review articles, etc. were excluded.

There was a specific focus on several key topics during the data extraction process. Among these topics were (1) carrier polymer; (2) loaded drug; (3) drug loading percentage, which is defined as the percentage of loaded drug mass relative to the total mass of system; (4) drug loading efficiency, which is defined as the percentage of the measured drug in the produced filament or 3D printed device relative to the theoretical loaded drug; (5) processing temperature; (6) drug loading approach; (7) 3D printer used; (8) intended application; and (9) produced samples.

## 3. Results and Discussion

### 3.1. Literature Search Output

**Figure 1:**
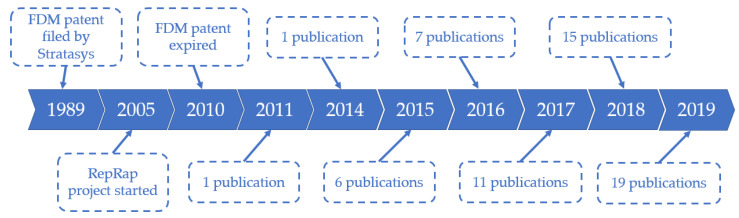
The number of publications included in this review over the years.

As shown in the Supplementary Material (Figure S1), the search engines resulted in 235 and 145 articles from WebofKnowledge and PubMed, respectively. The digital object identifier system was used to exclude duplicates from the results, which resulted in 267 articles. Finally, titles and abstracts were screened to remove irrelevant articles based on the criteria mentioned in the methods section. The excluded articles were 207, which revealed 60 articles to be included in this systematic review. Moreover, it was noted that the interest in this topic has increased over the years, as shown by an increase in the number of published articles. As demonstrated in Figure 1, only one paper was published in 2011, while 19 papers were published in 2019. All the data gathered from these publications are summarized in the Supplementary Material (Table S1).

### 3.2. Drug Loading Approaches

**Figure 2:**
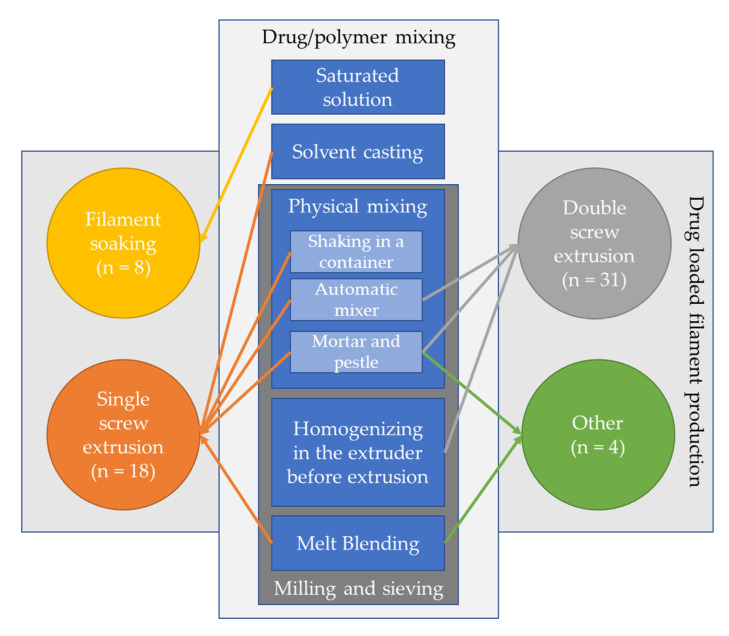
Summary of methods used for the mixing of polymer and drug prior to the filament preparation process with number of articles; arrows represent the mixing method used for each drug-loaded filament production approach. (Note: two methods of extrusion: a double screw extruder and a piston extruder were used by Kempin et al. [17].)

In all the selected articles in this review, the process for producing a drug-loaded medical device was conducted by preparing a filament which consists of carrier polymer/s and loaded drug/s. Subsequently, this filament is loaded into an FFF 3D printer by which the device is produced. According to the selected articles, an initial step might be required if the polymer’s form is incompatible with the drug. This is generally done when the polymer is supplied in the form of pellets or chips, while drugs are mostly supplied in a powdered form. In processes such as physical mixing, homogenizing in the extruder before extrusion, and melt-blending (see Figure 2), a large variation in the size between the polymer and the drug can be a major cause of an inhomogeneous drug distribution in the produced filament [16]. Thus, a pre-step of milling and sieving is required to reform the polymer or polymers. However, this step is not essential for other techniques such as solvent casting. This is because the polymer is typically dissolved in an organic solvent. On the other hand, the drug can be either dissolved or suspended. Subsequently, a stirring step is performed that enables the homogenization of the mixture. Finally, the last step includes the production of filaments. This can be done using different approaches, which will be discussed later in this review. The different steps and approaches are illustrated in Figure 2.

#### 3.2.1. Filament Soaking Approach

**Figure 3:**
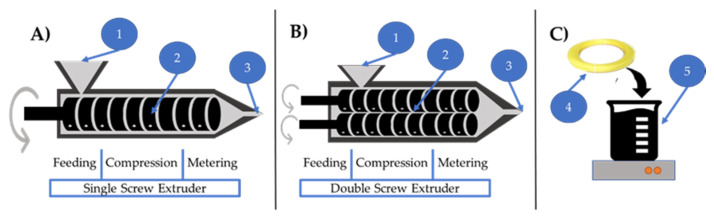
Illustration of drug loading approaches; single (**A**) and double (**B**) screw extrusion systems and filament soaking (**C**). Labels on the figure: (1) material input, (2) screw system, (3) filament output, (4) already prepared filament, and (5) solution/dispersion system.

**Figure 4:**
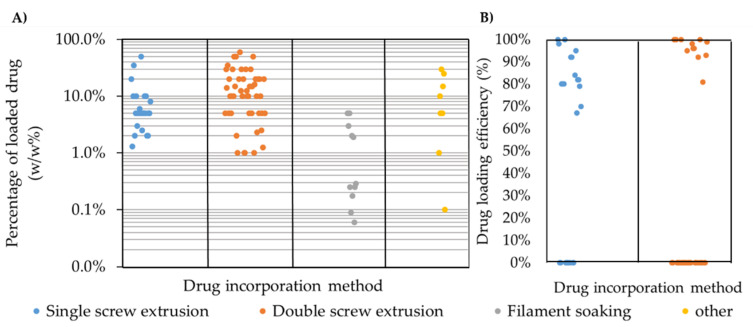
Percentage of drug loading (*w*/*w*) into polymer based on the drug incorporation method (**A**) and drug loading efficiency (**B**) as reported; zero values refer to publications that have not reported this value.

In the filament soaking approach (illustrated in Figure 3C), a saturated solution or dispersion containing the selected drug dissolved/dispersed in an appropriate solvent is prepared. Subsequently, the filament that contains the carrier polymer is soaked and conditioned in the prepared system for a certain period of time to assure diffusion into the polymeric matrix. It is worth noting that only one publication stated preparing a dispersion system [18], while all other publications that used this approach prepared a solution system. This method greatly depends on the swelling of the polymers when submerged in certain solvents. The time needed for proper swelling of the filament greatly affects the drug loading [19]. The soaking duration can vary between 12 h, as reported by Qamar et al. [20] up to 3–4 days, as reported by Ibrahim et al. [21]. In this process, a commercial filament can be used, which is the main advantage of this approach. However, this process has many drawbacks as it uses solvents that might be toxic. Additionally, the drug loading capacity is very limited and highly dependent on the diffusion kinetics between the drug and the polymeric matrix in the solvent/dispersion system. As seen in Figure 4A, the maximum drug loading capacity reported in the publications included in this review was 5% (*w*/*w*). This process is not eco-friendly due to the large portion of wasted drugs in the solution/dispersion. Moreover, the industrialization of this method can be relatively challenging.

#### 3.2.2. Single Screw Extruder

In the single screw extrusion configuration, as shown in Figure 3A, a single rotating screw is used to generate the flow of material. This screw contains three sections—(1) feeding, (2) compression, and (3) metering section [22]. The material is fed from a hopper in the feeding section. Then, it melts via shear stress and/or heating elements along the compression and metering sections. Finally, it flows out of a well-defined nozzle. In this approach, the drug and the carrier polymer should be sufficiently mixed before feeding to the extruder to assure optimum homogeneity of the output mixture. This is vital, as the single screw extrusion process provides limited homogenization of the mixture during extrusion. In addition, drugs are typically provided in a powdered form. However, polymers can exhibit several forms, ranging from small particles up to pellets or chips, which can significantly affect the homogeneity of the mixture [16]. Thus, an earlier step such as grinding and sieving, as illustrated in Figure 2, is required for obtaining the polymer in a suitable form. Once a suitable particle size is achieved, several mixing approaches were used in the reviewed articles, such as mixing in a container using an automatic mixer or mixing using a mortar and pestle. On the other hand, other approaches that do not require the polymer preparation stage, such as solvent casting or melt blending were used in other studies [23,24,25,26,27]. The former process includes the use of solvents to dissolve the polymer with the loaded drug. The latter process involves melting the polymer and homogenizing it with the drug before extrusion. The preparation of the physical mixture of the polymers and the drugs prior to extrusion is crucial as poor mixing could result in non-homogeneous blending, which will affect the content uniformity of the final product. An example of non-ideal extrusion was illustrated by Goyanes et al. [28] and Long et al. [29]. The drug loading in the extruded sample was around 80% and 70% of the theoretical value, respectively. This was due to adhesion of powdered drug particles to the walls of the container, including the hopper and the barrel during extrusion using a single screw extruder.

#### 3.2.3. Double Screw Extruder

In the double screw extruder, as shown in Figure 3B, a variety of configurations can be used. Generally, a co-rotating or counter-rotating double screw with an intermeshing or non-intermeshing setup is used [22]. This system has several advantages over the single screw extrusion system, such as an improved mixing that enables the production of a more homogeneous output, as illustrated by Patil et al. [30]. Additionally, lower shear stresses compared to single screw extruders are applied, which is essential during the extrusion of sensitive drugs. Another important advantage in this system is self-cleaning during extrusion. When the two screws are placed in an intermeshing configuration, the flight of one screw can remove the material in the root of the other one [6]. It was noted that 50% of the reviewed articles used this approach for the production of feedstock filaments due to its better mixing capabilities. Moreover, it was noted that some publications provided fewer details when describing the premixing method before extrusion, such as Isreb et al. [31], Sadia et al. [32], Okwuosa et al. [33,34], and Pietrzak et al. [35]. These publications described a melt mixing step in the extruder for around 5–10 min before starting the extrusion process. Additionally, Hollander et al. [36] used a similar melt mixing step before extrusion. However, this research group added 1/5 of the polymer amount, and then the drug and subsequently the remaining polymer to the extruder. Finally, a melt mixing step of 10 min was performed. This resulted in a drug loading efficiency of 83%, which was the lowest in this approach. During this mixing period, the extruder was set to temperatures suitable for melting the mixture. Moreover, other research groups used a mixer or mortar and pestle for homogenizing the mixture before feeding it to the extruder. Generally, the double screw extrusion configuration resulted in a drug loading efficiency ranging from 83% [36] to 100%. Furthermore, this process did not differ from the single screw extrusion in terms of drug loading capacity, as the maximum loading percentage reported among the reviewed articles was 60% (*w*/*w*), as described by Verstraete et al. [37].

#### 3.2.4. Other Approaches

Few articles used other processes for the preparation of the filaments. For producing drug-loaded filaments, one method used a piston extruder in which a piston was used to push the melted material through a well-defined nozzle. This technique was shown by Kempin et al. [17,38] and Teo et al. [27]. Another approach used a melt blender to create a homogenized blend of the polymer and the drug. Then the melt was cast in a tube with a desired diameter [39]. In these studies, the materials were premixed using a mortar and pestle or a mixer.

It was noted that more than 80% of the publications considered for this review used a single screw or double screw extruder to produce the filament for 3D printing. This is due to the well-established knowledge related to the HME process used in the pharmaceutical industries. Figure 4A summarizes the drug loading capacity based on the technology used, which was between 1% and 60% (*w*/*w*) in the single and double screw extrusion system. These two methods provide wide flexibility in terms of drug loading capacity, taking into consideration the limitation in drug homogeneity in the single screw extrusion process. A major cause of such poor homogeneity is the high pressure generated during extrusion, even when an ideal mixture is fed to the extruder. This pressure may compress the dispersed particles in the melt and can generate agglomerates which will affect the homogeneity of the mixture [6]. The filament soaking approach was discussed in several publications. However, it showed a limited capability in terms of the drug loading capacity, even with a long soaking duration, as demonstrated by Ibrahim et al. [21], in which the loading percentage ranged from 0.3% up to 5% (*w*/*w*). This could be due to the limited diffusion of drugs into the polymer matrix, as the diffusion is highly dependent on the polymer swelling. However, it should be noted that extensive swelling might cause significant deformation of the filament, which will result in inconsistencies in the diameter of the filament after drying. This could cause problems during 3D printing.

Filaments produced using other mixing approaches were mostly extruded using a piston extruder. This method has similar flexibility in the drug loading capacity to screw extrusion processes. However, the drug–polymer mixture homogeneity totally relies on the premixing process before the extrusion. This is due to the lack of material homogenizing during the extrusion process. Most of the publications included in this review that used a piston extruder reported that a homogenized system was prepared before the extrusion process via a pre-melt mixing step.

Figure 4B compares the drug loading efficiency in the single and double screw extrusion system. This comparison excluded the filament soaking approach, as most studies focused on reporting the percentage of drug that was loaded into the filament and not the loading efficiency. This is because the percentage of the remaining drug in the solution/dispersion system was not always discussed and the focus was only on measuring the percentage of the drug loaded into the filament. As shown in Figure 4B, 10 out of 18 and 13 out of 31 publications discussed the drug loading efficiency of the produced filaments using single screw or double screw extrusion, respectively. The variation in the drug loading efficiency reported in the single screw extrusion ranges between 65% and 100% of the total percentage of loaded drug. This significant variation can be due to poor mixing using this configuration. Furthermore, the homogeneity of the drug among the polymeric matrix mostly depends on the premixing method used prior to extrusion. Long et al. [29] directly extruded the polymer pellets and the drug using the single screw configuration. Subsequently, the produced filament was grinded and re-extruded. However, the drug loading efficiency in this approach ranged between 70% to 90%. Mixing the polymer and the drug in the powdered form using mortar and pestle, mixer or by manual shaking in a container yielded large variations in loading efficiency, as the process is highly subject to human error. This was observed by Gultekin et al. [40], Li et al. [41], and Goyanes et al. [28,42,43] where the drug loading efficiency was 96–100%, 88–96%, and 82–97%, respectively. On the other hand, smaller variations are observed in the double screw extrusion system, as the drug loading efficiency reported for double screw extruders ranged between 83% and 100%. The lower variation in drug loading efficiency demonstrated the superiority of homogenizing the mixture within a double screw extruder.

### 3.3. Carrier Polymer and Drugs Used

The carrier polymer plays two critical roles in the drug loading process. On the one hand, it is the medium in which the loaded drug is stored before it is released. On the other hand, it should provide the required mechanical properties to satisfy the requirements for extrusion and 3D printing. Moreover, the processing of this carrier polymer from raw material to end-product should not compromise the APIs’ activity. Selecting suitable temperatures for the process depends on many parameters, such as the physical properties, the melt rheology and the degradation temperatures of the ingredients due to the heat sensitivity of some drugs [44]. An example of a drug’s degradation during processing was reported by Goyanes et al. [26], in which salicylic acid was degraded during extrusion and the measured drug was only 30% of theoretical loaded drug. Moreover, it is vital to reach a suitable extrusion temperature to ensure the proper flow of material during filament preparation or 3D printing. Generally, temperatures needed during filament preparation are lower compared to 3D printing. This is due to the high torque achieved in a single or a double screw extrusion system. However, during 3D printing, a higher temperature is needed to achieve the required viscosity and ensure optimum material flow.

**Figure 5:**
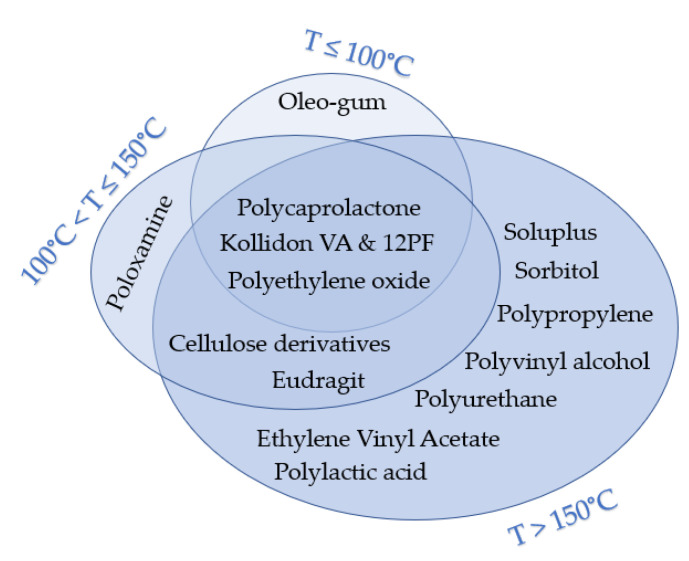
Carrier polymers used in the reviewed publications based on the maximum processing temperature used.

**Figure 6:**
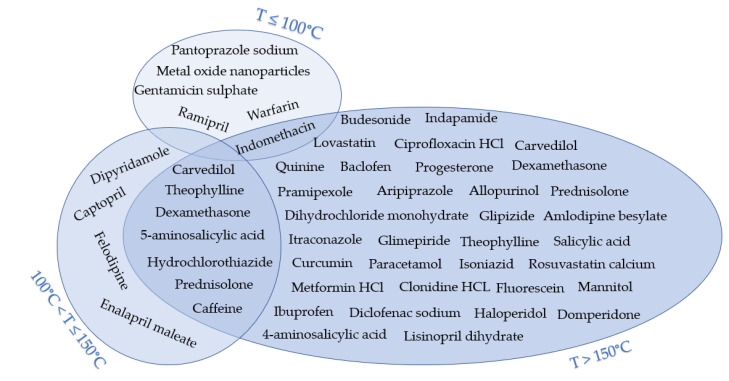
APIs used in the reviewed publications based on the maximum processing temperature used.

The processing temperatures observed in the publications considered for this review were classified into three categories, namely, (1) less than 100 °C, (2) between 100 °C and 150 °C, and (3) More than 150 °C. Figure 5 and Figure 6 illustrate the polymers used and APIs based on these three categories, respectively. Around 10% of the reviewed publications used temperatures below 100 °C. One study used oleo-gum resins from benzoin, which were loaded with metal oxide and 3D printed at 60 °C [45]. Concomitantly, another research group discussed the applicability of 3D printing at low temperatures to incorporate thermolabile drugs such as ramipril [46]. In this study, drug-loaded Kollidon^®^ VA64 and 12 PF were 3D printed at 90 °C. Moreover, other researchers used polyethylene glycol 6000 and Kollidon^®^ 12 PF to load pantoprazole sodium and 3D print at around 54 °C and 79 °C, respectively [17]. Furthermore, polycaprolactone was used to load gentamycin sulfate and print at a temperature of 100 °C [27]. Around 17% of publications used in this review used medium processing temperatures between 100 °C and 150 °C. Costa et al. [47] mixed poloxamine and polycaprolactone to load dexamethasone. The mixture was 3D printed at 110 °C. Sadia et al. [48] prepared a mixture of Eudragit^®^ E, triethyl citrate, and tri-calcium phosphate and used hydrochlorothiazide as the loaded drug and 3D printed the produced filament at 135 °C. In contrast, 73% of the reviewed publications performed printing at temperatures higher than 150 °C. The highest processing temperature was reported by Tagami et al. [39]. In this study, a polyvinyl alcohol filament was loaded with curcumin as a model drug, which was 3D printed at a temperature range of 150 °C to 250 °C. However, a change in color was reported in the produced samples as the processing temperature increased, which resulted in the degradation of the drug. The measured drug concentration was decreased to 79% (*w*/*w*) at 150 °C and 15% (*w*/*w*) at 250 °C. Moreover, in a study by Farto-Vaamonde et al. [49], prednisolone and dexamethasone were loaded into polylactic acid filaments using the filament soaking approach. Subsequently, the drug-loaded filaments were printed at 220 °C. Zhang et al. [50] investigated the possibility of coupling 3D printing with HME by testing different formulations of carrier polymers. Acetaminophen was used as a model drug in combination with several polymers, such as Benecel™ HPMC E5, Klucel™ HPC, Aqualon™ EC N14, Soluplus^®^, and Eudragit^®^ L100. Filaments from different formulations were extruded at a temperature range between 140 °C to 160 °C and 3D printed at 200 °C. In this study, no drug degradation was reported as 100% of the drug was released within 24 h.

The intended application is a key player in the selection of the carrier polymer for the drug-loaded device. Moreover, the properties of this polymer will determine the efficacy of the system. In some applications, such as orthopedics, high mechanical properties are required. Most often, a polymer with high mechanical properties, such as polylactic acid, requires high processing temperatures. This can be very challenging when the loaded drug is thermally labile.

Moreover, another challenge is the 3D printing process. Unlike HME, it is not a continuous process, this is due to repeated interruptions in the extrusion process during printing and non-printing movements. This could cause a longer exposure to high temperatures, which can lead to more degradation of the loaded drug. Thus, it is very important to take into account all the conditions that the drug will endure during the HME and the 3D printing process and to select a carrier polymer based on these considerations to ensure the efficacy of the drug-loaded system that is produced.

#### 3.4. 3D Printing of Drug-Loaded Devices

**Figure 7:**
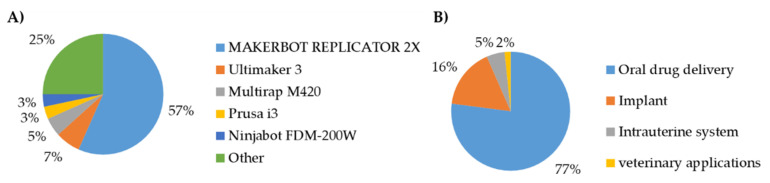
Printers’ manufacturer mentioned in the publications included (**A**) and summary of applications discussed among publications considered for this review (**B**).

After the expiration of the Stratasys patent [51] and the establishment of the Reprap open-source society [52], the number of fused filament fabrication 3D printers has rapidly increased. The relatively low price of these printers has enabled further research to investigate their applicability for producing drug-loaded medical devices. Figure 7A illustrates the 3D printers used in the selected articles. It was noted that the Makerbot Replicator 2X is the most widely used 3D printer, as more than 50% of the publications included in this review used it.

### 3.5. Intended Application

**Figure 8:**
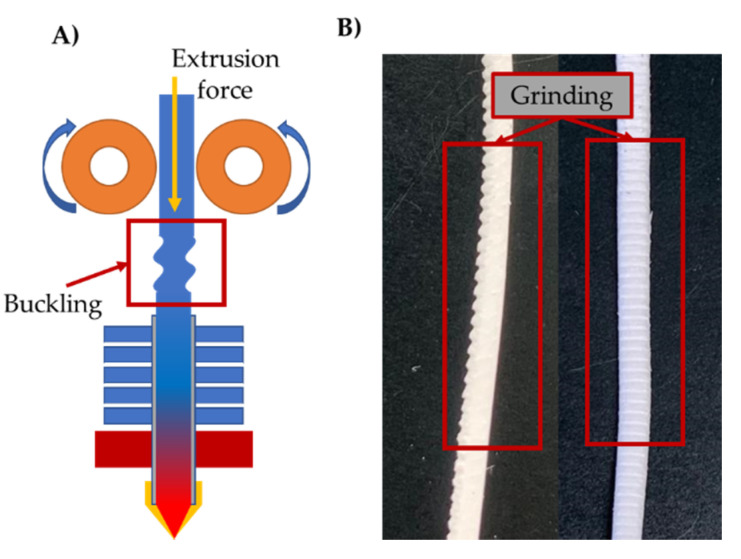
Buckling during filament extrusion (**A**) and grinding occurs on the filament during 3D printing (**B**).

A wide variety of applications were covered by the publications considered for this review. These applications are illustrated in Figure 7B and were divided into the following four categories: (1) oral drug delivery systems, (2) implants, (3) intrauterine systems, and (4) veterinary applications. More than 75% of publications included in this review investigated the production of drug-loaded devices for oral drug delivery. This is due to the well-established experience in HME processes to produce oral drug delivery systems over the past years. In order to produce a printable filament, certain physical properties should be met. One of the crucial parameters is the mechanical properties of these filaments to allow proper feeding into the FFF 3D printer. Thus, many researchers focused on conducting a formulation investigation and exploration, such as Illyes et al. [53], who investigated the printability of various formulations loaded with Carvedilol. Additionally, other research groups used design optimization approaches for formulation selection, such as quality by design and design of experiments such as Palekar et al. [54], Korte and Quodbach [11], and Nukala et al. [55]. The driving system in most FFF 3D printers relies on the un-melted filament to push the melted portion and maintain the material flow. Thus, the filament should have two main mechanical properties—(1) sufficient strength to avoid buckling and (2) high surface hardness to avoid filament grinding caused by the extruder gears, as shown in Figure 8A,B.

Moreover, many research groups screened formulations to determine the drug release profile, such as Arafat et al. [56], Goyanes et al. [57,58], Solanki et al. [59], Zhang et al. [50], Tagami et al. [39], Alhijjaj et al. [60], Skowyra et al. [61], and Yang et al. [62]. Other groups aimed to control the drug release profile to achieve sustained release, immediate release or a combination of both, such as Kimura et al. [63], Gultekin et al. [40], Isreb et al. [31], and Pietrzak et al. [35]. The geometry of the tablets was thoroughly investigated as it directly affects the release profile. Sadia et al. [48] investigated the production of channeled tablets to accelerate drug release. Tagami et al. [64] and Gioumouxouzis et al. [65] produced a defined drug release profile by printing a water-soluble drug-loaded material and water-insoluble material in different configurations. Scoutaris et al. [66] explored 3D printing of tablets made from a sweet chewable material designed to be palatable to children. Zhang et al. [67] investigated the correlation between the release profile and the tablet geometry using mathematical models to achieve zero-order release. Goyanes et al. [43] and Oblom et al. [68] determined the effects of different geometries, such as a cube, sphere, cylinder, pyramid and ring, and the size of the tablet on the release profile. Fuenmayor et al. [69] studied the difference in drug release profile between conventional tablet manufacturing approaches, such as compression molding and injection molding, and the new AM technology. In this study, the tablets manufactured by compression and injection molding showed immediate and sustained release profiles, respectively. However, tablets manufactured by FFF combined those two release profiles concomitantly. In general, 3D printing showed great potential for producing systems with tailored a release profile. This capability was achievable by optimizing the composition of the carrier polymer/s by combining water soluble and insoluble polymers. Additionally, varying the shape and geometry of the produced devices also determines the release rate of the drug. This was possible because 3D printing allows manufacturing geometries that are not achievable through other manufacturing processes. The surface area plays a key role in this approach for tailoring the release profile as needed.

Drug loading capacity was also investigated by Verstraete et al. [37] and Tidau et al. [70] as it is vital for determining the maximum drug that can be loaded into a 3D printed device. Furthermore, Chai et al. [71] and Jamroz et al. [72] improved the solubility of poor water soluble drugs using hot melt extrusion and investigated producing 3D printed tablets with these drugs.

Three publications studied the printing of intrauterine systems. Fu et al. [24] discussed the production of vaginal rings loaded with progesterone using a blend of polylactic acid and polycaprolactone. Moreover, Hollander et al. [36] and Genina et al. [73] discussed the production of T-shaped intrauterine systems loaded with Indomethacin. Active pharmaceutical ingredients were loaded at different weight percentages and different release profiles were achieved. One article discussed the production of drug-loaded projectiles for veterinary application. Long et al. [29] discussed the possibility of using 3D printing to develop progesterone loaded devices in the shape of a projectile. The aim was to investigate the applicability of producing drug-loaded ballistic devices for drug delivery in wildlife.

Ten articles discussed the production of implantable devices. Kempin et al. [25] and Genina et al. [73] produced model implants using 3D printing technology for selecting and optimizing the formulations of drug-loaded polymers. Additionally, they studied the produced material properties such as printability, release profile and mechanical performance. Farto-Vaamonde et al. [49] and Costa et al. [47] worked on producing drug-loaded scaffolds for tissue engineering and regeneration medicine. Qamar et al. [20] and Teo et al. [27] produced drug-loaded meshes for eradicating bacterial infections. Horst et al. [45] discussed producing disks for testing antibacterial activity of oleo-gum-resin loaded with nano-oxides. Eleftheriadis et al. [74] and Jiang et al. [23] worked on producing drug-loaded devices for oral applications such as mucoadhesive buccal films and an orthodontic retainer, respectively. Goyanes et al. [26] discussed utilizing 3D scanning for producing a 3D model of a personalized implant and then 3D printing it with a drug-loaded polymeric material for anti-acne applications. It can be noted that there was only a limited focus on producing drug-loaded implantable devices. However, the interest in these forms is increasing as there was only one publication in 2010 compared to three in 2019. Additionally, with the continuous improvement of the FFF process, it is expected to gain more attention in the coming years.

## 4. Conclusions

This systematic review discussed the drug incorporation approaches used for preparing filaments for FFF 3D printing. A survey of 60 publications showed the differences between the preparation techniques of filaments used in FFF 3D printing. Three methods were most widely used—(1) single screw extruder, (2) double screw extruder, and (3) filament soaking. Fewer articles used other mixing approaches (6% of the total review articles). The majority of the reviewed articles (50%) used double screw extruders to prepare the feedstock filaments. This system was capable of loading a high percentage of API with suitable filament homogeneity and minimal preparation efforts before extrusion. The single screw extrusion system was the second most popular (30%), showing the capability of high drug loading of around 50% (*w*/*w*). However, due to the limitation in producing homogenous mixtures, it is vital to properly mix the materials prior to the extrusion step. On the other hand, the filament soaking approach was the third most popular option (13%), and it is relatively simple as commercially available filaments can be used. However, it is very limited in terms of drug loading capacity. Additionally, a substantial portion of waste byproduct is produced with this approach.

A wide variety of polymers were used in the studies; however, it was noted that the most significant limiting factor in choosing the material was the processing temperature. As most APIs are thermally labile, it is vital to stay below degradation limits. Moreover, filaments should show suitable mechanical and rheological properties to allow sufficient feeding and printing performance. The intended application for the majority of the reviewed articles was producing oral drug delivery systems. Other research groups discussed applications such as implant, intrauterine systems, and veterinary applications.

## 5. Future Perspectives

AM technologies show high potential for personalized medicine. Its added value can be classified into two categories—(1) dose and formulation flexibility and (2) new possibilities in the produced geometries. On the one hand, the concept of “production on demand” can be applied when formulating using AM technologies. Thus, such technologies provide great potential for producing patient-specific formulations with relatively low cost. On the other hand, coupled with 3D scanning technologies, patient-specific devices can be produced to match the shape needed in a customized manner. Moreover, the opportunity for creating different geometries can help to tailor the drug release profile as required. Additionally, small-scale batch production using AM is relatively cheap compared to conventional manufacturing technologies.

The typical approach for producing drug-loaded devices that consist of filament preparation followed by 3D printing using FFF 3D printers has been explored intensively. However, this approach has some limitations due to the need for the HME step to prepare filaments with the intended diameter. Additionally, the shelf-life stability of such filaments is crucial for a smooth feeding performance and consistent quality for the end-product. Some filaments are hygroscopic, which might negatively affect their mechanical properties. Thus, another trend was introduced by a few research groups in which the raw materials were directly fed into the printer. Recently, research groups such as Fanous et al. [75], Ong et al. [76], and Goyanes et al. [77] discussed this approach. However, it is critical to understand that raw material homogeneity is the most important factor in this process. Fanous et al. [75] used a piston extruder in the 3D printing process that does not offer any mixing abilities during printing. On the other hand, Ong et al. [76] and Goyanes et al. [77] used a single screw extruder that has minimal capacity for mixing and homogenizing. Moreover, Justino Netto and Silveira [78] illustrated a prototype of a 3D printer fitted with a double screw extruder. However, testing and validating the suitability of such extrusion systems are still needed.

## Supplementary Materials

The following are available online at https://www.mdpi.com/1999-4923/12/6/517/s1. Figure S1: Flowchart diagram of the publications selection process conducted for this systematic review, Table S1: Summary of the data gathered from publications considered in this systematic review.
