# Supplementary material for: Production of Drug Delivery Systems Using Fused Filament Fabrication: A Systematic Review
Source: Pharmaceutics. 2020 Jun 5;12(6):517. doi: 10.3390/pharmaceutics12060517 (PMC7356885; doi:10.3390/pharmaceutics12060517)
Supplement: Supplementary file 1 [file pharmaceutics-12-00517-s001.pdf]

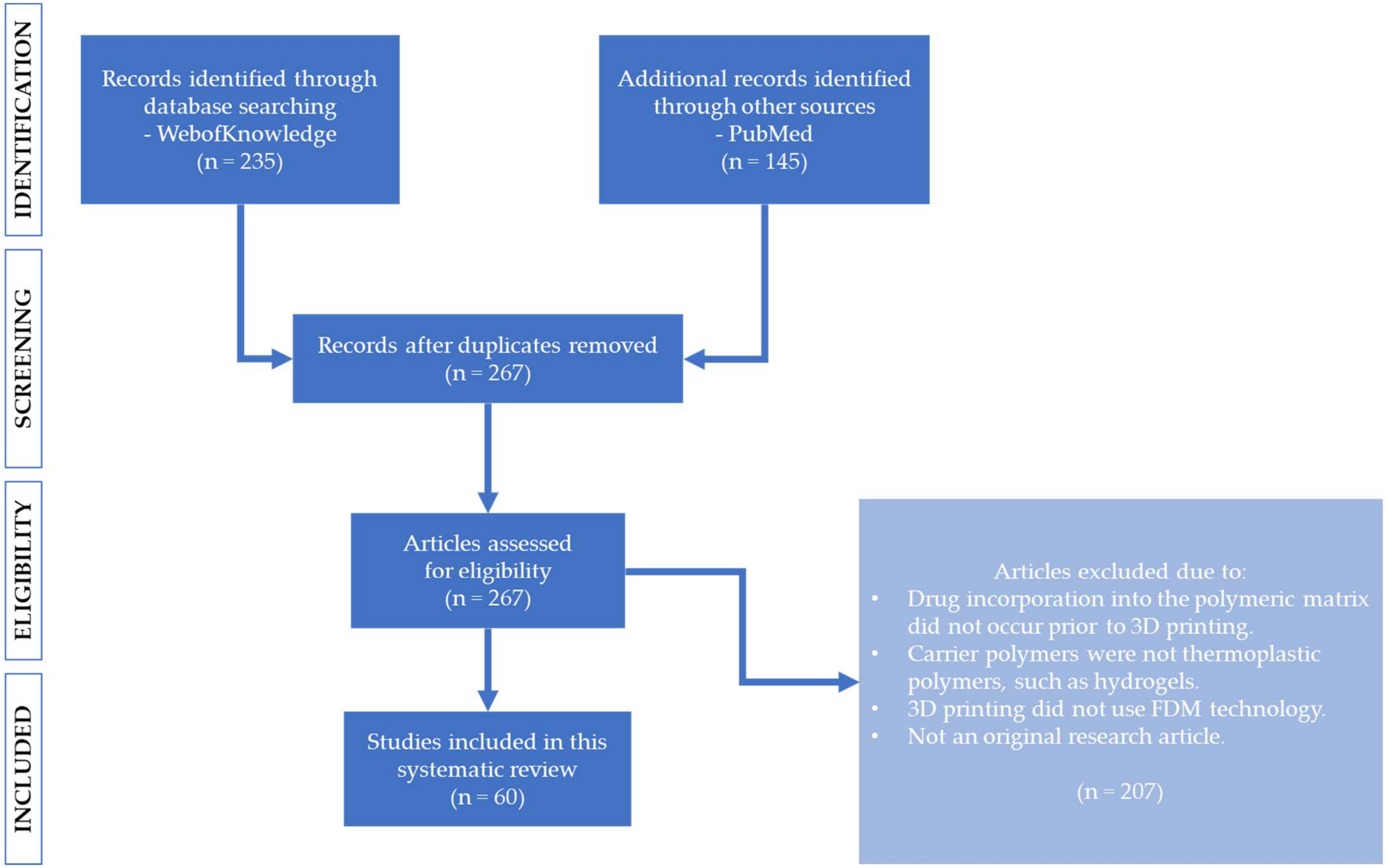

Figure S1. Flowchart diagram of the publications selection process conducted for this systematic review.

Table S1: Summary of the data gathered from publications considered in this systematic review.

Carrier polymer	Physical form	Loaded drug	Drug loading amount	Drug loading approach	Processing temperature	3D printer used	Intended application	Dosage form	Ref.
Polycaprolactone (Capa 6506)<br><br>Kollidon VA64<br><br>Polyethylene oxide (Mw=300,000)	Powder	Lovastatin<br><br>Hydrochlorothiazide	5% (w/w)	Stage 1: Sieved at 250 µm meshes<br><br>Stage 2: Physically mixed for 15 minutes<br><br>Stage 3: HME using a twin extruder<br>Milled and sieved at 250 µm, mixed for 15 minutes<br><br>Note: the process was repeated twice.	HME: 140°C<br>3D printing:160°C	MakerGear M2	Oral drug delivery	Tablets	[1]
Polyvinyl alcohol	Filament	Curcumin	5% (w/w)	Stage 1: N/A<br><br>Stage 2: A saturated dispersion was prepared and, in some cases, heated<br><br>Stage 3: The filament was soaked in this solution.	3D printing:210°C	Ninjabot FDM-200W	Oral drug delivery	Tablets	[2]
Polypropylene<br><br>Polyvinyl alcohol	Filament	Ciprofloxacin HCl	3% (w/w) for PP<br>5% (w/w) for PVA	Stage 1: N/A<br><br>Stage 2: A saturated solution was prepared and, in some cases, heated and stirred or sonicated.<br><br>Stage 3: The filament was soaked in this solution for 12 or 24 hours.	3D printing:190°C (PP), 200°C (PVA)	MAKERBOT REPLICATOR 2X	Meshes, treatment of hernia	Implant	[3]

Polylactic acid (2002D)<br><br>Polycaprolactone (MW: 80,000)<br><br>Polyethylene glycol (4000)	Pellets, powder	Clonidine hydrochloride	1% (w/w)	Stage 1: N/A<br><br>Stage 2: Drug and polymers were melt-mixed in a heated water bath<br><br>Stage 3: A single screw extruder was used to produce a filament for 3D printing	HME: 190°C<br>3D printing: 190°C	Manli Technology Group CF-12410B	Orthodontic Retainer	Implant	[4]
Hydroxypropyl cellulose<br><br>Polyvinylpyrrolidone	Powder	Itraconazole	20% (w/w)<br><br>All formulations were loaded with efficiency of 100%	Stage 1: N/A<br><br>Stage 2: Drug and polymers were physically blended in a bag for 5 minutes<br><br>Stage 3: A single screw extruder was used to produce the filament for 3D printing	HME: 135°C<br>3D printing: 180°C	3D Magix MF-2200D	Oral drug delivery	Tablets	[5]
Eudragit EPO<br><br>POLYOX™ WSR N10<br><br>POLYOX™ WSR N80	Powder	Pramipexole dihydrochloride monohydrate	1.3% (w/w)<br><br>All formulations were loaded with efficiency of 96-101%	Stage 1: N/A<br><br>Stage 2: Drug and polymer were mixed in mixer for 10 minutes.<br><br>Stage 3: A single screw extruder was used to produce a filament for 3D printing	HME: 120-130°C<br>3D printing: 160-175°C	MAKERBOT REPLICATOR 2X	Oral drug delivery	Tablets	[6]
Polylactic acid (Mw: 150,556)	Filament	Prednisolone<br><br>Dexamethasone	0.25% (w/w) for prednisolone<br>0.09% (w/w) for dexamethasone	Stage 1: N/A<br><br>Stage 2: A saturated solution was prepared.<br><br>Stage 3: The filament was soaked in the solution for 24 hours at 37°C in an oscillator.	3D printing: 220°C	REGEMAT 3D V1	Tissue regeneration	Implant, Cylindrical scaffold	[7]

Polyvinyl alcohol	Filament	Metformin HCl	2% (w/w)	Stage 1: N/A<br><br>Stage 2: A saturated solution was prepared.<br><br>Stage 3: The filament was soaked in this solution for 3 to 4 days.	3D printing: 205°C	MAKERBOT REPLICATOR 2X	Oral drug delivery	Tablets	[8]
Polyethylene oxides (Mw: 100K, 200K, 300K, 600K, 900K)<br><br>Polyethylene glycol (Mw: 6K)	Powder	Theophylline	30% (w/w)<br><br>All formulations were loaded with efficiency of 100%	Stage 1: N/A<br><br>Stage 2: Drug and the polymer were melt-mixed in a twin screw extruder for 5 minutes.<br><br>Stage 3: The mixed materials were extruded to produce filament for 3D printing	HME: 60-80°C<br>3D printing: 105-145°C	MAKERBOT REPLICATOR 2X	Oral drug delivery	Tablets	[9]
Polyvinyl alcohol**,** Ultimaker filament	Filament	Ciprofloxacin hydrochloride	10%-35% (w/w)	Stage 1: The filament was crushed to produce pellets/particle ranging from 5000-250 µm<br><br>Stage 2: Drug and polymer were physically mixed in a balloon and in some cases a Dibutyl Sebacate was used to improve adhesion.<br><br>Stage 3: The mixture was extruded using a single screw extruder to produce filament for 3D printing.	HME: 160-175°C<br>3D printing: 195°C	Ultimaker 3	Oral drug delivery	Tablets	[10]

AFFINISOLTM HPMC HME 15lv<br><br>CARBOWAXTM (PEG 8000)<br><br>Polyethylene oxide (Mw: 100,000), PEO-L (Mw:2,000,000)	Powder	Theophylline	14%-35% (w/w)<br><br>Drug loading efficacy was around 100%	Stage 1: N/A<br><br>Stage 2: Drug and polymer were physically mixed.<br><br>Stage 3: A twin screw extruder was used to produce filament for 3D printing	HME: 120-170°C<br>3D printing: 110-210°C	Ultimaker 3	Oral drug delivery	Tablets	[11]
Polycaprolactone (Capa 6506)<br><br>Kollidon VA64<br><br>Polyethylene oxide (Mw: 300,000)	Powder	Caffeine	5% (w/w)<br><br>Drug loading efficacy was around 100%	Stage 1: Polymer were sieved with a 450 µm mesh.<br><br>Stage 2: Drug and polymer were mixed using a mixer for 15 minutes at 50 rpm.<br><br>Stage 3: A twin extruder was used to produce a filament for 3D printing.	HME: 80-140°C<br>3D printing: 150°C	MAKERBOT REPLICATOR 2X	Oral drug delivery	Tablets	[12]
Hydroxypropyl methylcellulose<br><br>Affinisol HME 15LV<br><br>Kollidon SR<br><br>Eudragit E PO (EPO)<br><br>hydroxypropyl cellulose	Powder	Carvedilol	20% (w/w)	Stage 1: N/A<br><br>Stage 2: Drug and polymers were mixed in mortar and pestle.<br><br>Stage 3: A twin extruder was used to produce filament for 3D printing	HME: 130 C<br>3D printing: 135-200 C	CraftBot Plus	Oral drug delivery	Tablets	[13]

Parteck MXP<br><br>Sorbitol (Parteck SI 150),	Powder	Baclofen	10% (w/w)	Stage 1: N/A<br><br>Stage 2: Drug and polymers were mixed using a mortar and pestle and then physically blended using a mixer.<br><br>Stage 3: A twin screw extruder was used to produce filament for 3D printing	HME: 160°C<br>3D printing: 190°C	MAKERBOT REPLICATOR 2X	Oral drug delivery	Tablets	[14]
Parteck MXP<br><br>Sorbitol (Parteck SI 150)<br><br>Hydroxypropyl cellulose (Klucel)<br><br>Kollidon VA64<br><br>Affinisol™15LV	Powder	Metformin hydrochloride	5%, 10% (w/w)	Stage 1: N/A<br><br>Stage 2: Drug and polymer were mixed using mortar and pestle and then physically blended using a mixer.<br><br>Stage 3: A twin screw extruder was used to produce filament for 3D printing	HME: 140-170°C<br>3D printing: 200°C	MAKERBOT REPLICATOR 2X	Oral drug delivery	Tablets	[15]
Hydroxypropylmethylcellulose (Benecel E5, K100M)<br><br>Hydroxypropylcellulose (Klucel EF, HF)<br><br>Polyethylene oxide (Sentry Polyox WSR N-80 NF, Sentry™ Polyox™ WSR N-750 NF)<br><br>Eudragit® RS PO, RL PO and L 100	Powder	Isoniazid	30% (w/w)<br><br>Drug loading efficiency was around 100%	Stage 1: N/A<br><br>Stage 2: Drug and polymers were physically blended using a mixer.<br><br>Stage 3: A twin screw extruder was used to produce filament for 3D printing	HME: 100-155°C<br>3D printing: 165-195°C	MAKERBOT REPLICATOR 2X	Oral drug delivery	Tablets	[16]

Polyethylene glycol (Mw: 6000)<br><br>Polyvinylpyrrolidone (k-value 12)<br><br>Polycaprolactone (Mw: 14,000)<br><br>Cellulose acetate phthalate (CAP)<br><br>Eudragit L100-55<br><br>Hydroxypropyl methyl cellulose phthalate	Powder	Pantoprazole sodium	10% (w/w)	Stage 1: N/A<br><br>Stage 2: Drug and polymer were mixed using mortar and pestle.<br><br>Stage 3: Filament for 3D printing was producing using two methods: 1) A twin extruder was used and 2) a self-made piston extruder.	HME: 50-130°C<br>3D printing: 50-160°C	Multirap M420	Oral drug delivery	Tablets	[17]
Eudragit EPO	Powder	Hydrochlorothiazide<br><br>Enalapril maleate	0%-50% (w/w) for hydrochlorothiazide 15% (w/w) for enalapril maleate	Stage 1: N/A<br><br>Stage 2: Drug and polymer were mixed using mortar and pestle.<br><br>Stage 3: A twin extruder was using to produce filament for 3D printing	HME: 100°C<br>3D printing: 135°C	MAKERBOT REPLICATOR 2X	Oral drug delivery	Tablets	[18]
polylactic acid (2003D grade)	Pellets	Progesterone	2%, 5% and 10% (w/w)<br><br>Drug loading efficiency was 70-90%	Stage 1: Before the second extrusion, filament was cut into pieces and grinded.<br><br>Stage 2: In the first extrusion, drug and polymer pellets were physically mixed.<br><br>Stage 3: A single screw extruder was used in the two extrusion steps to produce filament for 3D printing.	HME: 160°C<br>3D printing: 200°C	UP Mini 3D printer	Veterinary applications	Projectile contains contraceptive progesterone	[19]

PVA (Mowiol 4–88)<br><br>Eudragit RL PO<br><br>PLA (Resomer L 210 S)<br><br>PEG 400	Powder	Metformin HCl<br><br>Glimepiride	50% (w/w) for metformin<br>2% (w/w) for glimepiride	Glimepiride-loaded PVA<br>Stage 1: Polymer was sieved and grinded<br><br>Stage 2: Drug and polymer were mixed using mortar and pestle.<br><br>Stage 3: A twin extruder was used to produce filament for 3D printing.<br><br>Metformin-loaded Eudragit:<br>Stage 1: N/A<br><br>Stage 2: Melt mixing during extrusion.<br><br>Stage 3: A two-step extrusion process was used. First, using a single screw extruder and then using a twin screw extruder to produce filament for 3D printing.	HME: 160-190°C<br>3D printing: 170-205°C	MAKERBOT REPLICATOR 2X	Oral drug delivery	Tablets	[20]
Kollidon VA64, 12PF<br><br>PEG 1500	Powder	Ramipril	3% (w/w)<br><br>Drug loading efficiency was around 100%	Stage 1: N/A<br><br>Stage 2: Drug and polymer were mixed using mortar and pestle.<br><br>Stage 3: A single screw extruder was used to produce filament for 3D printing.	HME: 70°C<br>3D printing: 90°C	MAKERBOT REPLICATOR 2X	Oral drug delivery	Tablets	[21]

Eudragit EPO	Powder	Warfarin	1% (w/w)<br><br>Drug loading efficiency was around 90-100%	Stage 1: N/A<br><br>Stage 2: Drug and polymer were melt-mixed in the extruder for 5 minutes before extrusion.<br><br>Stage 3: A twin screw extruder were used for producing a filament for 3D printing.	HME: 90-100°C<br>3D printing: 135°C	MAKERBOT REPLICATOR 2X	Oral drug delivery	Tablets	[22]
Polyethylene glycol (Mw: 6000, 20000)<br><br>Poloxamer 407<br><br>Polyvinylpyrrolidone (k-value 12)<br><br>Kollidon VA64<br><br>Kollicoat IR<br><br>Kollidon CL	Powder	Pantoprazole sodium sesquihydrate	5%-30% (w/w)<br><br>Drug loading efficiency was around 90-100%	Stage 1: N/A<br><br>Stage 2: Drug and polymer were mixed using mortar and pestle for 3 minutes.<br><br>Stage 3: The mixture was extruded using a self-constructed piston extruder	HME: 41-145°C<br>3D printing: 45-87°C	Multirap M420	Oral drug delivery	Tablets	[23]
Gohsenol EG-05P<br><br>PLA	Powder	Fluorescein	Not mentioned	Stage 1: N/A<br><br>Stage 2: N/A<br><br>Stage 3: A twin extruder was used to produce filament for 3D printing	HME: 90-210°C<br>3D printing:190°C	Ultimaker 3	Oral drug delivery	Tablets	[24]

Polylactic acid (2002D)<br><br>Polycaprolactone (Mw: 80,000)<br><br>Polyethylene glycol 4000	Powder, pellets	Progesterone	5% (w/w)	Stage 1: N/A<br><br>Stage 2: Drug and polymer were melt-mixed and then cut into pieces for extrusion.<br><br>Stage 3: A single screw extruder was used to produce filament for 3D printing.	HME: 190°C<br>3D printing:195°C	CF-12410B, Manli Technology Group	Intrauterine system	Vaginal rings	[25]
Polyethylene glycol<br><br>Hypromellose acetate succinate	Powder	Indomethacin	20% (w/w)<br><br>weight and content uniformity variation of 5–10%	Stage 1: N/A<br><br>Stage 2: Drug and polymer were mixed using a mixer for 10 minutes at 100 rpm.<br><br>Stage 3: A twin extruder was used to produce filament for 3D printing.	HME: 40-140°C<br>3D printing:165°C	Airwolf3D HD2xR	Oral drug delivery	Tablets	[26]
Tecoflex™ (EG-72D, EG-80A)<br><br>TecophilicTM (SP-60D-60, SP-93A-100, TG-2000)	Powder, pellets	Theophylline anhydrous<br><br>Metformin hydrochloride	0%-60% (w/w)	Stage 1: N/A.<br><br>Stage 2: Drug and polymer were mixed.<br><br>Stage 3: A twin extruder was used to produce filament for 3D printing.	HME: 100-180°C<br>3D printing: 120-180°C	MAKERBOT REPLICATOR 2X	Oral drug delivery	Tablets	[27]

Eudragit E	Powder	Hydrochlorothiazide	12.5% (w/w)	Stage 1: N/A<br><br>Stage 2: Drug and polymer were melt-mixed using the extruder before extrusion for 5 minutes.<br><br>Stage 3: A twin extruder was used to produce filament for 3D printing.	HME: 90-100°C<br>3D printing: 135°C	MAKERBOT REPLICATOR 2X	Oral drug delivery	Tablets	[28]
Eudragit VR RL PO<br><br>Polyethylene glycol 4000	Powder	Anhydrous theophylline	30% (w/w)<br><br>Drug loading efficiency was around 97-99%	Stage 1: Drug and polymer were sieved using a 355 µm mesh.<br><br>Stage 2: Drug and polymer were mixed using a mixer for 20 minutes.<br><br>Stage 3: A twin screw extruder was used to produce filament for 3D printing.	HME: 30-175°C<br>3D printing: 180°C	Prodim XXL Pro	Oral drug delivery	Tablets	[29]
Kollidon VA64<br><br>Kollicoat IR<br><br>Affinisol 15cP<br><br>Aqoat AS-MG	Powder	Haloperidol	10%, 20% (w/w)<br><br>Drug loading efficiency was around 95-97%	Stage 1: N/A<br><br>Stage 2: Drug and polymer were mixed using a mixer.<br><br>Stage 3: A twin extruder was used to produce filament for 3D printing.	HME: 150°C<br>3D printing: 210°C	MAKERBOT REPLICATOR 2X	Oral drug delivery	Tablets	[30]

Benecel HPMC E5<br><br>Soluplus®	Powder	Paracetamol	1% (w/w)	Stage 1: N/A<br><br>Stage 2: Drug and polymer were mixed in a mixer at 25 rpm for 30 minutes.<br><br>Stage 3: A twin extruder was used to produce filament for 3D printing.	HME: 160°C<br>3D printing: 200°C	Ultimaker 3	Oral drug delivery	Tablets	[31]
Poval 4–88	Powder	Aripiprazole	0.55 (mg/cm^2)	Stage 1: N/A<br><br>Stage 2: Drug and polymer were mixed.<br><br>Stage 3: A single screw extruder was used to produce filament for 3D printing	HME: 172°C<br>3D printing: 190°C	Zmorph 2.0S	Oral drug delivery	Orodispersible films	[32]
Mowiol 4-88<br><br>Polylactic acid	Powder	Carvedilol<br><br>Hydrochlorothiazide<br><br>Mannitol	6% (w/w)	Stage 1: Polymer was sieved and grinded using 850 µm mesh.<br><br>Stage 2: Drug and polymer were mixed.<br><br>Stage 3: A single screw extruder were used to produce filament for 3D printing.	HME: 170°C<br>3D printing: 200-220°C	MAKERBOT REPLICATOR 2X	Oral drug delivery	Tablets	[33]
Hydroxypropylmethylcellulose acetate succinate HPMCAS LG<br><br>HPMCAS MG<br><br>HPMCAS HG (Aqoat)	Powder	Paracetamol	5%-50% (w/w)	Stage 1: N/A<br><br>Stage 2: Drug and polymer were mixed using a mortar and pestle.<br><br>Stage 3: A single screw extruder was used to produce filament for 3D printing.	HME: 80-110°C<br>3D printing: 180-190°C	MAKERBOT REPLICATOR 2X	Oral drug delivery	Tablets	[34]

Polyvinyl alcohol (YiShengInc)	Filament	Glipizide	2.5%, 5% (w/w)<br><br>Drug loading efficiency was around 88-96%	Stage 1: Filament was grinded until become in powder form.<br><br>Stage 2: Drug and polymer were mixed using mortar and pestle.<br><br>Stage 3: A single screw extruder was used to produce filament for 3D printing.	HME: 180°C<br>3D printing: 195°C	Clouovo Delta-MK2	Oral drug delivery	Tablets	[35]
Hydroxypropyl cellulose (Klucel TM)	Powder	Domperidone	1% (w/w)	Stage 1: N/A<br><br>Stage 2: Drug and polymer were mixed using mortar and pestle.<br><br>Stage 3: A twin extruder was used to produce filament for 3D printing.	HME: 145-150°C<br>3D printing: 210°C	MakerBot Replicator 2X	Oral drug delivery	Tablets	[36]
Eudragit RS<br><br>Poly(L-lactide) (Resomer L206S)<br><br>Polycaprolactone (Mw: 14,000)<br><br>Ethyl cellulose (ETHOCEL Standard 45 Premium)	Powder	Quinine	5% (w/w)	Stage 1: N/A<br><br>Stage 2: Drug and Polymer was mixed using solvent casting.<br><br>Stage 3: A single screw extruder was used to produce filament for 3D printing.	HME: 47-140°C<br>3D printing: 53-164°C	Multirap M420	Drug delivery system	Implant (hollow cylinder)	[37]
Benecel HPMC E5<br><br>Klucel HPC EF, LF<br><br>Aqualon EC N14<br><br>Soluplus<br><br>Eudragit1 L100	Powder	Paracetamol	30% (w/w)	Stage 1: N/A<br><br>Stage 2: Drug and polymer were mixed in a mixer for 20 minutes at 25 rpm.<br><br>Stage 3: A twin screw extruder was used to produce filament for 3D printing.	HME: 140-160°C<br>3D printing: 200°C	Prusa i3	Oral drug delivery	Tablets	[38]

PVA (Nippon Syntheti)	Filament	Curcumin	1.75 (mg/g) in the filament	Stage 1: N/A<br><br>Stage 2: A saturated solution was prepared.<br><br>Stage 3: The filament was soaked in the solution over night at room temperature.	3D printing: 150-250°C	Ninjabot FDM-200W	Oral drug delivery	Tablets	[39]
Oleo-gum-resins from benzoin, myrrha, olibanum	Powder	Metal oxide nanoparticles (TiO_2, Cu_2O, and MoO_3)	10% (w/w)	Stage 1: N/A<br><br>Stage 2: Drug and polymer were mixed.<br><br>Stage 3: A single screw extruder was used to produce filament for 3D printing.	HME: 70-85°C<br>3D printing: 80°C	Prusa i3	Bacterial infection prevention	Implant, disks	[40]
PVA (Makerbot Inc)	Filament	Paracetamol<br><br>Caffeine	5%, 10% (w/w)<br><br>Drug loading efficiency was around 82-86% and 94-95% for paracetamol and caffeine, respectively	Stage 1: Filament was cut into small pieces and grinded.<br><br>Stage 2: Drug and polymer were mixed using mortar and pestle and a shaker-mixer.<br><br>Stage 3: A single screw extruder was used to produce filament for 3D printing.	HME: 180°C<br>3D printing: 200°C	MAKERBOT REPLICATOR 2X	Oral drug delivery	Tablets	[41]

Eudragit EPO	Powder	Theophylline<br><br>5-ASA<br><br>Captopril<br><br>Prednisolone	12.5% (w/w)<br><br>Drug loading efficiency was around 88-96%	Stage 1: N/A<br><br>Stage 2: Drug and polymer were melt-mixed using the extruder before extrusion for 5 minutes.<br><br>Stage 3: A twin extruder was used to produce filament for 3D printing.	HME: 90-100°C<br>3D printing: 135°C	MAKERBOT REPLICATOR 2X	Oral drug delivery	Tablets	[42]
Polyethylene glycol (Mw: 4000)<br><br>Polyethylene Oxide (POLYOX WSR N10 LEO)<br><br>Eudragit EPO	Powder	Felodipine	10% (w/w)	Stage 1: N/A<br><br>Stage 2: Drug and polymer were mixed using mortar and pestle for 2 minutes.<br><br>Stage 3: A twin screw extruder was used to produce filament for 3D printing.	HME: 100-130°C<br>3D printing: 150°C	MAKERBOT REPLICATOR 2X	Oral drug delivery	Tablets	[43]
Polyvinylpyrrolidone (Mw: 40,000)	Powder	Theophylline<br><br>Dipyridamole	10% (w/w)<br><br>Drug loading efficiency was around 100%	Stage 1: N/A<br><br>Stage 2: Drug and polymer were melt-mixed using the extruder before extrusion for 5 minutes.<br><br>Stage 3: A twin extruder was used to produce filament for 3D printing.	HME: 90-100°C<br>3D printing: 110°C	MAKERBOT REPLICATOR 2X	Oral drug delivery	Tablets	[44]

PCL (CAPA 6500)	Filament	Indomethacin	5%, 15%, 30% (w/w)<br><br>Drug loading efficiency was 73-90%	Stage 1: N/A<br><br>Stage 2: Drug and polymer were melt-mixed using the extruder before extrusion for 10 minutes.<br><br>Stage 3: A twin extruder was used to produce filament for 3D printing.	HME: 100°C<br>3D printing: 100°C	MakerBot Replicator 2X	Intrauterine system	T-shaped	[45]
EVA copolymer (ATEVA 1070, 1075A, 1081G, 1241, 1641, 1821A, 1850A, 1880A, 2821A, 3325A)<br><br>PCL (CAPA™ 6500)	Filament	Indomethacin	5%, 15% (w/w)	Stage 1: N/A<br><br>Stage 2: Drug and polymer were melt-mixed using the extruder before extrusion for 10 minutes.<br><br>Stage 3: A twin extruder was used to produce filament for 3D printing.	HME: 105-120°C<br>3D printing: 100-215°C	MakerBot Replicator 2X	Intrauterine system	T-shaped, subcutaneo us rods	[46]
Flex EcoPLA BLUE 45D<br><br>Polycaprolactone (Mw: 80,000)	Filament	Salicylic acid	2% (w/w)<br><br>Drug loading efficiency was around 67%	Stage 1: N/A<br><br>Stage 2: Drug and Polymer was mixed using solvent casting.<br><br>Stage 3: A single screw extruder was used to produce filament for 3D printing.	HME: 60-190°C<br>3D printing: 170-230°C	MakerBot Replicator 2X	Nose shape, anti-acne drug loaded device	Implant	[47]

Polyvinyl alcohol	Filament	Budesonide	2%, 5% (w/w)<br><br>Drug loading efficiency was around 82%	Stage 1: Filament was cut into small pieces and grinded.<br><br>Stage 2: Drug and polymer were mixed using mortar and pestle and a shaker-mixer.<br><br>Stage 3: A single screw extruder was used to produce filament for 3D printing.	HME: 170°C<br>3D printing: 190°C	MakerBot Replicator 2X	Oral drug delivery	Tablets	[48]
Polyvinyl alcohol	Filament	Paracetamol	5% (w/w)<br><br>Drug loading efficiency was around 79%	Stage 1: Filament was cut into small pieces and grinded.<br><br>Stage 2: Drug and polymer were mixed using mortar and pestle and a shaker-mixer.<br><br>Stage 3: A single screw extruder was used to produce filament for 3D printing.	HME: 180°C<br>3D printing: 180°C	MakerBot Replicator 2X	Oral drug delivery	Cube, pyramid, cylinder, sphere and torus	[49]
Eudragit RL100, RS100<br><br>Hydroxypropyl cellulose (SSL)	Powder	Theophylline	50% (w/w)<br><br>Drug loading efficiency was around 91-95%	Stage 1: N/A<br><br>Stage 2: Drug and polymer were melt-mixed using the extruder before extrusion for 5 minutes.<br><br>Stage 3: A twin extruder was used to produce filament for 3D printing.	HME: 110-130°C<br>3D printing: 140-170°C	MakerBot Replicator 2X	Oral drug delivery	Tablets	[50]

Polyvinyl alcohol	Filament	Prednisolone	1.9% (w/w)	Stage 1: N/A<br><br>Stage 2: A saturated solution was prepared.<br><br>Stage 3: The filament was soaked in the solution for 24 hours at 30 C.	3D printing: 230°C	MakerBot Replicator 2X	Oral drug delivery	Tablets	[51]
polyvinyl alcohol	Filament	5-aminosalicylic acid<br><br>4-aminosalicylic acid	0.06% and 0.25% (w/w) for the 5-ASA and 4-ASA, respectively	Stage 1: N/A<br><br>Stage 2: A dispersion of drug in a solvent was prepared.<br><br>Stage 3: The filament was soaked in the solution for 24 hours under stirring.	3D printing: 210°C	MakerBot Replicator 2X	Oral drug delivery	Tablets	[52]
polyvinyl alcohol	Filament	Fluorescein	0.29% (w/w)	Stage 1: N/A<br><br>Stage 2: A solution of drug in a solvent was prepared.<br><br>Stage 3: The filament was soaked in the solution for 24 hours under stirring.	3D printing: 220°C	MakerBot Replicator 2X	Oral drug delivery	Tablets	[53]
PCL (Mw: 80,000)	Pellets	Gentamicin sulphate	5%, 15%, 25% (w/w)	Stage 1: N/A<br><br>Stage 2: Materials were melt-mixed.<br><br>Stage 3: A piston extruder was used to produce filament for 3D printing.	3D printing: 100°C	Stratasys 3D Modeler RP system	Bacterial infection prevention	Implant, 3-dimensional mesh with honeycomb-like pattern	[54]

PVA (PVA05, PVA18, Gohsenol EG 05P, EG 18P)<br><br>Eudragit RL100, RS100	Powder	Allopurinol	1% (w/w)<br><br>Drug loading efficiency was around 98-99%	Stage 1: materials were milled and sieved using 250 µm mesh after mixing.<br><br>Stage 2: Drug and polymer were mixed using mortar and pestle.<br><br>Stage 3: A twin screw extruder was used to produce filament for 3D printing.	HME: 175-200°C<br>3D printing: 200°C	KLONER3D 240TWIN	Oral drug delivery	Structure with different shapes	[55]
Ethyl cellulose<br><br>Hydroxypropyl methylcellulose (K100-LV Premium)	Powder	Ibuprofen	16-20% (w/w)	Stage 1: N/A<br><br>Stage 2: Drug and polymer were mixed.<br><br>Stage 3: A twin extruder was used to produce filament for 3D printing.	HME: 100-120°C<br>3D printing: 170-186°C	A3 JGAURORA	Oral drug delivery	Structure with different shapes	[56]
Polyvinylpyrrolidone (Mw: 40,000)<br><br>PEG400	Powder	Theophylline<br><br>Budesonide<br><br>Diclofenac sodium	10%, 2.3%, 20% (w/w)<br><br>Drug loading efficiency was around 85-99%	Stage 1: N/A<br><br>Stage 2: Drug and polymer were melt-mixed using the extruder before extrusion for 5 minutes.<br><br>Stage 3: A twin extruder was used to produce filament for 3D printing.	HME: 100-135°C<br>3D printing: 110-185°C	MAKERBOT REPLICATOR 2X	Oral drug delivery	Tablets	[57]

Poloxamine 908 (Tetronic)<br><br>Polycaprolactone (Mw: 50,000)	Powder	Dexamethasone	0.001%, 0.1% (w/w)	Stage 1: Polymer were grinded before use.<br><br>Stage 2: Polymers were mixed in a turbula for 15 minutes. Drug and the mixer were melt-mixed<br><br>Stage 3: The mixture was poured in a tube with a diameter of a filament and placed into and ice bath to solidify the mixture.	HME: 80°C<br>3D printing: 110°C	Printrbot Simple	Bone regeneration	Implant, Scaffold	[58]
Mowiol 4-88<br><br>Chitosan	Powder	Diclofenac sodium	8% (w/w)<br><br>Drug loading efficiency was around 60-100%	Stage 1: N/A<br><br>Stage 2: Drug and polymer were mixed.<br><br>Stage 3: A single screw extruder was used to produce filament for 3D printing.	HME: 165-169°C<br>3D printing: 190-200°C	Makerbot Replicator 2X	Oral drug delivery	Films	[59]
Sorbitol<br><br>Parteck MXP	Powder	Lisinopril dihydrate<br><br>Indapamide<br><br>Amlodipine besylate<br><br>Rosuvastatin calcium	5%-20%, 10%-2.5%, 5%-1.25% and 20%-5% for Lisinopril dihydrate, Indapamide, Amlodipine besylate and Rosuvastatin calcium, respectively<br><br>Drug loading efficiency was around 93-99%	Stage 1: N/A<br><br>Stage 2: Drug and polymer were mixed for 5 minutes at 100 rpm.<br><br>Stage 3: A twin extruder was used to produce filament for 3D printing.	HME: 100°C<br>3D printing: 210°C	Makerbot Replicator 2X	Oral drug delivery	Tablets	[60]

References

1. Fuenmayor, E.; O'Donnell, C.; Gately, N.; Doran, P.; Devine, D.M.; Lyons, J.G.; McConville, C.; Major, I. Mass-customization of oral tablets via the combination of 3D printing and injection molding. *International Journal of Pharmaceutics* **2019**, *569*, 118611, doi:10.1016/j.ijpharm.2019.118611.
2. Tagami, T.; Kuwata, E.; Sakai, N.; Ozeki, T. Drug Incorporation into Polymer Filament Using Simple Soaking Method for Tablet Preparation Using Fused Deposition Modeling. *Biological and Pharmaceutical Bulletin* **2019**, *42*, 1753–1760, doi:10.1248/bpb.b19-00482.
3. Qamar, N.; Abbas, N.; Irfan, M.; Hussain, A.; Arshad, M.S.; Latif, S.; Mehmood, F.; Ghori, M.U. Personalized 3D printed ciprofloxacin impregnated meshes for the management of hernia. *Journal of Drug Delivery Science and Technology* **2019**, *53*, 101164, doi:10.1016/j.jddst.2019.101164.
4. Jiang, H.; Fu, J.; Li, M.; Wang, S.; Zhuang, B.; Sun, H.; Ge, C.; Feng, B.; Jin, Y. 3D-Printed Wearable Personalized Orthodontic Retainers for Sustained Release of Clonidine Hydrochloride. *AAPS PharmSciTech* **2019**, *20*, 260, doi:10.1208/s12249-019-1460-6.
5. Kimura, S.; Ishikawa, T.; Iwao, Y.; Itai, S.; Kondo, H. Fabrication of Zero-Order Sustained-Release Floating Tablets *via* Fused Depositing Modeling 3D Printer. *Chemical and Pharmaceutical Bulletin* **2019**, *67*, 992–999, doi:10.1248/cpb.c19-00290.
6. Gültekin, H.E.; Tort, S.; Acartürk, F. An Effective Technology for the Development of Immediate Release Solid Dosage Forms Containing Low-Dose Drug: Fused Deposition Modeling 3D Printing. *Pharm Res* **2019**, *36*, 128, doi:10.1007/s11095-019-2655-y.
7. Farto-Vaamonde, X.; Auriemma, G.; Aquino, R.P.; Concheiro, A.; Alvarez-Lorenzo, C. Post-manufacture loading of filaments and 3D printed PLA scaffolds with prednisolone and dexamethasone for tissue regeneration applications. *European Journal of Pharmaceutics and Biopharmaceutics* **2019**, *141*, 100–110, doi:10.1016/j.ejpb.2019.05.018.
8. Ibrahim, M.; Barnes, M.; McMillin, R.; Cook, D.W.; Smith, S.; Halquist, M.; Wijesinghe, D.; Roper, T.D. 3D Printing of Metformin HCl PVA Tablets by Fused Deposition Modeling: Drug Loading, Tablet Design, and Dissolution Studies. *AAPS PharmSciTech* **2019**, *20*, 195, doi:10.1208/s12249-019-1400-5.
9. Isreb, A.; Baj, K.; Wojsz, M.; Isreb, M.; Peak, M.; Alhnan, M.A. 3D printed oral theophylline doses with innovative 'radiator-like' design: Impact of polyethylene oxide (PEO) molecular weight. *International Journal of Pharmaceutics* **2019**, *564*, 98–105, doi:10.1016/j.ijpharm.2019.04.017.
10. Saviano, M.; Aquino, R.P.; Del Gaudio, P.; Sansone, F.; Russo, P. Poly(vinyl alcohol) 3D printed tablets: The effect of polymer particle size on drug loading and process efficiency. *International Journal of Pharmaceutics* **2019**, *561*, 1–8, doi:10.1016/j.ijpharm.2019.02.025.
11. Tidau, M.; Kwade, A.; Finke, J.H. Influence of High, Disperse API Load on Properties along the Fused-Layer Modeling Process Chain of Solid Dosage Forms. *Pharmaceutics* **2019**, *11*, 194, doi:10.3390/pharmaceutics11040194.
12. Fuenmayor, E.; Forde, M.; Healy, A.V.; Devine, D.M.; Lyons, J.G.; McConville, C.; Major, I. Comparison of fused-filament fabrication to direct compression and injection molding in the manufacture of oral tablets. *International Journal of Pharmaceutics* **2019**, *558*, 328–340, doi:10.1016/j.ijpharm.2019.01.013.
13. Ilyés, K.; Kovács, N.K.; Balogh, A.; Borbás, E.; Farkas, B.; Casian, T.; Marosi, G.; Tomuță, I.; Nagy, Z.K. The applicability of pharmaceutical polymeric blends for the fused deposition modelling (FDM) 3D technique: Material considerations–printability–process modulation, with consecutive effects on in vitro release, stability and degradation. *European Journal of Pharmaceutical Sciences* **2019**, *129*, 110–123, doi:10.1016/j.ejps.2018.12.019.
14. Palekar, S.; Nukala, P.K.; Mishra, S.M.; Kipping, T.; Patel, K. Application of 3D printing technology and quality by design approach for development of age-appropriate pediatric formulation of baclofen. *International Journal of Pharmaceutics* **2019**, *556*, 106–116, doi:10.1016/j.ijpharm.2018.11.062.
15. Nukala, P.K.; Palekar, S.; Patki, M.; Patel, K. Abuse Deterrent Immediate Release Egg-Shaped Tablet (Egglets) Using 3D Printing Technology: Quality by Design to Optimize Drug Release and Extraction. *AAPS PharmSciTech* **2019**, *20*, 80, doi:10.1208/s12249-019-1298-y.
16. Öblom, H.; Zhang, J.; Pimparade, M.; Speer, I.; Preis, M.; Repka, M.; Sandler, N. 3D-Printed Isoniazid Tablets for the Treatment and Prevention of Tuberculosis—Personalized Dosing and Drug Release. *AAPS PharmSciTech* **2019**, *20*, 52, doi:10.1208/s12249-018-1233-7.
17. Kempin, W.; Domsta, V.; Brecht, I.; Semmling, B.; Tillmann, S.; Weitschies, W.; Seidlitz, A. Development of a dual extrusion printing technique for an acid- and thermo-labile drug. *European Journal of Pharmaceutical Sciences* **2018**, *123*, 191–198, doi:10.1016/j.ejps.2018.07.041.

18. Sadia, M.; Isreb, A.; Abbadi, I.; Isreb, M.; Aziz, D.; Selo, A.; Timmins, P.; Alhnan, M.A. From ‘fixed dose combinations’ to ‘a dynamic dose combiner’: 3D printed bi-layer antihypertensive tablets. *European Journal of Pharmaceutical Sciences* **2018**, *123*, 484–494, doi:10.1016/j.ejps.2018.07.045.
19. Long, J.; Nand, A.V.; Ray, S.; Mayhew, S.; White, D.; Bunt, C.R.; Seyfoddin, A. Development of customised 3D printed biodegradable projectile for administrating extended-release contraceptive to wildlife. *International Journal of Pharmaceutics* **2018**, *548*, 349–356, doi:10.1016/j.ijpharm.2018.07.002.
20. Gioumouxouzis, C.I.; Baklavaridis, A.; Katsamenis, O.L.; Markopoulou, C.K.; Bouropoulos, N.; Tzetzis, D.; Fatouros, D.G. A 3D printed bilayer oral solid dosage form combining metformin for prolonged and glimepiride for immediate drug delivery. *European Journal of Pharmaceutical Sciences* **2018**, *120*, 40–52, doi:10.1016/j.ejps.2018.04.020.
21. Kollamaram, G.; Croker, D.M.; Walker, G.M.; Goyanes, A.; Basit, A.W.; Gaisford, S. Low temperature fused deposition modeling (FDM) 3D printing of thermolabile drugs. *International Journal of Pharmaceutics* **2018**, *545*, 144–152, doi:10.1016/j.ijpharm.2018.04.055.
22. Arafat, B.; Qinna, N.; Cieszynska, M.; Forbes, R.T.; Alhnan, M.A. Tailored on demand anti-coagulant dosing: An in vitro and in vivo evaluation of 3D printed purpose-designed oral dosage forms. *European Journal of Pharmaceutics and Biopharmaceutics* **2018**, *128*, 282–289, doi:10.1016/j.ejpb.2018.04.010.
23. Kempin, W.; Domsta, V.; Grathoff, G.; Brecht, I.; Semmling, B.; Tillmann, S.; Weitschies, W.; Seidlitz, A. Immediate Release 3D-Printed Tablets Produced Via Fused Deposition Modeling of a Thermo-Sensitive Drug. *Pharm Res* **2018**, *35*, 124, doi:10.1007/s11095-018-2405-6.
24. Tagami, T.; Nagata, N.; Hayashi, N.; Ogawa, E.; Fukushige, K.; Sakai, N.; Ozeki, T. Defined drug release from 3D-printed composite tablets consisting of drug-loaded polyvinylalcohol and a water-soluble or water-insoluble polymer filler. *International Journal of Pharmaceutics* **2018**, *543*, 361–367, doi:10.1016/j.ijpharm.2018.03.057.
25. Fu, J.; Yu, X.; Jin, Y. 3D printing of vaginal rings with personalized shapes for controlled release of progesterone. *International Journal of Pharmaceutics* **2018**, *539*, 75–82, doi:10.1016/j.ijpharm.2018.01.036.
26. Scoutaris, N.; Ross, S.A.; Douroumis, D. 3D Printed “Starmix” Drug Loaded Dosage Forms for Paediatric Applications. *Pharm Res* **2018**, *35*, 34, doi:10.1007/s11095-017-2284-2.
27. Verstraete, G.; Samaro, A.; Grymonpré, W.; Vanhoorne, V.; Van Snick, B.; Boone, M.N.; Hellemans, T.; Van Hoorebeke, L.; Remon, J.P.; Vervaet, C. 3D printing of high drug loaded dosage forms using thermoplastic polyurethanes. *International Journal of Pharmaceutics* **2018**, *536*, 318–325, doi:10.1016/j.ijpharm.2017.12.002.
28. Sadia, M.; Arafat, B.; Ahmed, W.; Forbes, R.T.; Alhnan, M.A. Channelled tablets: An innovative approach to accelerating drug release from 3D printed tablets. *Journal of Controlled Release* **2018**, *269*, 355–363, doi:10.1016/j.jconrel.2017.11.022.
29. Korte, C.; Quodbach, J. Formulation development and process analysis of drug-loaded filaments manufactured via hot-melt extrusion for 3D-printing of medicines. *Pharmaceutical Development and Technology* **2018**, *23*, 1117–1127, doi:10.1080/10837450.2018.1433208.
30. Solanki, N.G.; Tahsin, M.; Shah, A.V.; Serajuddin, A.T.M. Formulation of 3D Printed Tablet for Rapid Drug Release by Fused Deposition Modeling: Screening Polymers for Drug Release, Drug-Polymer Miscibility and Printability. *J Pharm Sci* **2018**, *107*, 390–401, doi:10.1016/j.xphs.2017.10.021.
31. Zhang, J.; Yang, W.; Vo, A.Q.; Feng, X.; Ye, X.; Kim, D.W.; Repka, M.A. Hydroxypropyl methylcellulose-based controlled release dosage by melt extrusion and 3D printing: Structure and drug release correlation. *Carbohydrate Polymers* **2017**, *177*, 49–57, doi:10.1016/j.carbpol.2017.08.058.
32. Jamróz, W.; Kurek, M.; Łyszczarz, E.; Szafraniec, J.; Knapik-Kowalczuk, J.; Syrek, K.; Paluch, M.; Jachowicz, R. 3D printed orodispersible films with Aripiprazole. *International Journal of Pharmaceutics* **2017**, *533*, 413–420, doi:10.1016/j.ijpharm.2017.05.052.
33. Gioumouxouzis, C.I.; Katsamenis, O.L.; Bouropoulos, N.; Fatouros, D.G. 3D printed oral solid dosage forms containing hydrochlorothiazide for controlled drug delivery. *Journal of Drug Delivery Science and Technology* **2017**, *40*, 164–171, doi:10.1016/j.jddst.2017.06.008.
34. Goyanes, A.; Fina, F.; Martorana, A.; Sedough, D.; Gaisford, S.; Basit, A.W. Development of modified release 3D printed tablets (printlets) with pharmaceutical excipients using additive manufacturing. *International Journal of Pharmaceutics* **2017**, *527*, 21–30, doi:10.1016/j.ijpharm.2017.05.021.
35. Li, Q.; Wen, H.; Jia, D.; Guan, X.; Pan, H.; Yang, Y.; Yu, S.; Zhu, Z.; Xiang, R.; Pan, W. Preparation and investigation of controlled-release glipizide novel oral device with three-dimensional printing. *International Journal of Pharmaceutics* **2017**, *525*, 5–11, doi:10.1016/j.ijpharm.2017.03.066.

36. Chai, X.; Chai, H.; Wang, X.; Yang, J.; Li, J.; Zhao, Y.; Cai, W.; Tao, T.; Xiang, X. Fused Deposition Modeling (FDM) 3D Printed Tablets for Intragastric Floating Delivery of Domperidone. *Sci Rep* **2017**, *7*, 1–9, doi:10.1038/s41598-017-03097-x.
37. Kempin, W.; Franz, C.; Koster, L.-C.; Schneider, F.; Bogdahn, M.; Weitschies, W.; Seidlitz, A. Assessment of different polymers and drug loads for fused deposition modeling of drug loaded implants. *European Journal of Pharmaceutics and Biopharmaceutics* **2017**, *115*, 84–93, doi:10.1016/j.ejpb.2017.02.014.
38. Zhang, J.; Feng, X.; Patil, H.; Tiwari, R.V.; Repka, M.A. Coupling 3D printing with hot-melt extrusion to produce controlled-release tablets. *International Journal of Pharmaceutics* **2017**, *519*, 186–197, doi:10.1016/j.ijpharm.2016.12.049.
39. Tagami, T.; Fukushige, K.; Ogawa, E.; Hayashi, N.; Ozeki, T. 3D Printing Factors Important for the Fabrication of Polyvinylalcohol Filament-Based Tablets. *Biological and Pharmaceutical Bulletin* **2017**, *40*, 357–364, doi:10.1248/bpb.b16-00878.
40. Horst, D.J.; Tebcherani, S.M.; Kubaski, E.T.; de Almeida Vieira, R. Bioactive Potential of 3D-Printed Oleo-Gum-Resin Disks: B. papyrifera, C. myrrha, and S. benzoin Loading Nanooxides—TiO2, P25, Cu2O, and MoO3 Available online: https://www.hindawi.com/journals/bca/2017/6398167/ (accessed on Nov 11, 2019).
41. Goyanes, A.; Kobayashi, M.; Martínez-Pacheco, R.; Gaisford, S.; Basit, A.W. Fused-filament 3D printing of drug products: Microstructure analysis and drug release characteristics of PVA-based caplets. *International Journal of Pharmaceutics* **2016**, *514*, 290–295, doi:10.1016/j.ijpharm.2016.06.021.
42. Sadia, M.; Sośnicka, A.; Arafat, B.; Isreb, A.; Ahmed, W.; Kelarakis, A.; Alhnan, M.A. Adaptation of pharmaceutical excipients to FDM 3D printing for the fabrication of patient-tailored immediate release tablets. *International Journal of Pharmaceutics* **2016**, *513*, 659–668, doi:10.1016/j.ijpharm.2016.09.050.
43. Alhijjaj, M.; Belton, P.; Qi, S. An investigation into the use of polymer blends to improve the printability of and regulate drug release from pharmaceutical solid dispersions prepared via fused deposition modeling (FDM) 3D printing. *European Journal of Pharmaceutics and Biopharmaceutics* **2016**, *108*, 111–125, doi:10.1016/j.ejpb.2016.08.016.
44. Okwuosa, T.C.; Stefaniak, D.; Arafat, B.; Isreb, A.; Wan, K.-W.; Alhnan, M.A. A Lower Temperature FDM 3D Printing for the Manufacture of Patient-Specific Immediate Release Tablets. *Pharm Res* **2016**, *33*, 2704–2712, doi:10.1007/s11095-016-1995-0.
45. Holländer, J.; Genina, N.; Jukarainen, H.; Khajeheian, M.; Rosling, A.; Mäkilä, E.; Sandler, N. Three-Dimensional Printed PCL-Based Implantable Prototypes of Medical Devices for Controlled Drug Delivery. *Journal of Pharmaceutical Sciences* **2016**, *105*, 2665–2676, doi:10.1016/j.xphs.2015.12.012.
46. Genina, N.; Holländer, J.; Jukarainen, H.; Mäkilä, E.; Salonen, J.; Sandler, N. Ethylene vinyl acetate (EVA) as a new drug carrier for 3D printed medical drug delivery devices. *European Journal of Pharmaceutical Sciences* **2016**, *90*, 53–63, doi:10.1016/j.ejps.2015.11.005.
47. Goyanes, A.; Det-Amornrat, U.; Wang, J.; Basit, A.W.; Gaisford, S. 3D scanning and 3D printing as innovative technologies for fabricating personalized topical drug delivery systems. *Journal of Controlled Release* **2016**, *234*, 41–48, doi:10.1016/j.jconrel.2016.05.034.
48. Goyanes, A.; Chang, H.; Sedough, D.; Hatton, G.B.; Wang, J.; Buanz, A.; Gaisford, S.; Basit, A.W. Fabrication of controlled-release budesonide tablets via desktop (FDM) 3D printing. *International Journal of Pharmaceutics* **2015**, *496*, 414–420, doi:10.1016/j.ijpharm.2015.10.039.
49. Goyanes, A.; Robles Martinez, P.; Buanz, A.; Basit, A.W.; Gaisford, S. Effect of geometry on drug release from 3D printed tablets. *International Journal of Pharmaceutics* **2015**, *494*, 657–663, doi:10.1016/j.ijpharm.2015.04.069.
50. Pietrzak, K.; Isreb, A.; Alhnan, M.A. A flexible-dose dispenser for immediate and extended release 3D printed tablets. *European Journal of Pharmaceutics and Biopharmaceutics* **2015**, *96*, 380–387, doi:10.1016/j.ejpb.2015.07.027.
51. Skowyra, J.; Pietrzak, K.; Alhnan, M.A. Fabrication of extended-release patient-tailored prednisolone tablets via fused deposition modelling (FDM) 3D printing. *European Journal of Pharmaceutical Sciences* **2015**, *68*, 11–17, doi:10.1016/j.ejps.2014.11.009.
52. Goyanes, A.; Buanz, A.B.M.; Hatton, G.B.; Gaisford, S.; Basit, A.W. 3D printing of modified-release aminosalicylate (4-ASA and 5-ASA) tablets. *European Journal of Pharmaceutics and Biopharmaceutics* **2015**, *89*, 157–162, doi:10.1016/j.ejpb.2014.12.003.
53. Goyanes, A.; Buanz, A.B.M.; Basit, A.W.; Gaisford, S. Fused-filament 3D printing (3DP) for fabrication of tablets. *International Journal of Pharmaceutics* **2014**, *476*, 88–92, doi:10.1016/j.ijpharm.2014.09.044.

54. Teo, E.Y.; Ong, S.-Y.; Khoon Chong, M.S.; Zhang, Z.; Lu, J.; Moochhala, S.; Ho, B.; Teoh, S.-H. Polycaprolactone-based fused deposition modeled mesh for delivery of antibacterial agents to infected wounds. *Biomaterials* **2011**, *32*, 279–287, doi:10.1016/j.biomaterials.2010.08.089.
55. Melocchi, A.; Uboldi, M.; Inverardi, N.; Briatico-Vangosa, F.; Baldi, F.; Pandini, S.; Scalet, G.; Auricchio, F.; Cerea, M.; Foppoli, A.; et al. Expandable drug delivery system for gastric retention based on shape memory polymers: Development via 4D printing and extrusion. *International Journal of Pharmaceutics* **2019**, *571*, 118700, doi:10.1016/j.ijpharm.2019.118700.
56. Yang, Y.; Wang, H.; Li, H.; Ou, Z.; Yang, G. 3D printed tablets with internal scaffold structure using ethyl cellulose to achieve sustained ibuprofen release. *European Journal of Pharmaceutical Sciences* **2018**, *115*, 11–18, doi:10.1016/j.ejps.2018.01.005.
57. Okwuosa, T.C.; Pereira, B.C.; Arafat, B.; Cieszynska, M.; Isreb, A.; Alhnan, M.A. Fabricating a Shell-Core Delayed Release Tablet Using Dual FDM 3D Printing for Patient-Centred Therapy. *Pharm Res* **2017**, *34*, 427–437, doi:10.1007/s11095-016-2073-3.
58. Costa, P.F.; Puga, A.M.; Díaz-Gomez, L.; Concheiro, A.; Busch, D.H.; Alvarez-Lorenzo, C. Additive manufacturing of scaffolds with dexamethasone controlled release for enhanced bone regeneration. *International Journal of Pharmaceutics* **2015**, *496*, 541–550, doi:10.1016/j.ijpharm.2015.10.055.
59. Eleftheriadis, G.K.; Ritzoulis, C.; Bouropoulos, N.; Tzetzis, D.; Andreadis, D.A.; Boetker, J.; Rantanen, J.; Fatouros, D.G. Unidirectional drug release from 3D printed mucoadhesive buccal films using FDM technology: In vitro and ex vivo evaluation. *European Journal of Pharmaceutics and Biopharmaceutics* **2019**, *144*, 180–192, doi:10.1016/j.ejpb.2019.09.018.
60. Pereira, B.C.; Isreb, A.; Forbes, R.T.; Dores, F.; Habashy, R.; Petit, J.-B.; Alhnan, M.A.; Oga, E.F. 'Temporary Plasticiser': A novel solution to fabricate 3D printed patient-centred cardiovascular 'Polypill' architectures. *European Journal of Pharmaceutics and Biopharmaceutics* **2019**, *135*, 94–103, doi:10.1016/j.ejpb.2018.12.009.
